# Discordant serum lipid parameters

**DOI:** 10.1186/s12944-021-01445-5

**Published:** 2021-02-11

**Authors:** Ozge Kurmus, Turgay Aslan, Murat Eren, Kursat Akbuga

**Affiliations:** Department of Cardiology, Faculty of Medicine, Ufuk University, Mevlana Bulvari (Konya yolu), No: 86-88, Balgat, Ankara, Turkey

Dear Editor,

We read with interest the paper by Fonseca et al. entitled “Apolipoprotein B and non-high-density lipoprotein cholesterol reveal a high atherogenicity in individuals with type 2 diabetes and controlled low-density lipoprotein-cholesterol” [1]. The authors state that 22% of the diabetic patients with target low-density lipoprotein cholesterol (LDL-C) level had non-high-density lipoprotein cholesterol (non-HDL-C) level above the target level. Target levels were defined according to the European Society of Cardiology/European Atherosclerosis Society 2016 Guideline for the Management of Dyslipidemia [2].

We previously demonstrated that in 574 consecutive patients who underwent coronary angiography, 15% of them had discordance between LDL-C and non-HDL-C levels [3]; 30% of our study group had type 2 diabetes mellitus (T2DM). In our study, patients with a high difference between non-HDL-C and LDL-C levels were more commonly females, had T2DM and high triglyceride levels [3]. Also, they less commonly received statin therapy [3]. Both the current study [1] and our study [3] measured fasting blood lipids. Measuring apolipoprotein B (Apo B) and oxidized LDL-C level is one of the advantages of the Fonseca et al. study [1]. The authors reported that in addition to non-HDL-C, Apo B and oxidized LDL-C levels were above the recommended range in 25 and 44% of the patients with controlled LDL-C levels, respectively. Non-HDL-C, Apo B and oxidized LDL-C are all potential atherogenic lipid particles [4, 5]. The findings of Fonseca et al. [1] add significant information to previously published data. Comparing the characteristics of patients with non-HDL-C, Apo B and oxidized LDL-C below target with non-HDL-C, Apo B and oxidized LDL-C above target in groups within and above target LDL-C levels separately would be useful for better understanding which patient might have discordant lipid parameters.

Although the size of the study populations and the cut-off values for LDL-C and non-HDL-C to classify patients differ between the current study [1] and our study [3], we both pointed out that sizeable proportion of patients may have low LDL-C and high non-HDL-C levels; T2DM seems to be a risk factor for this pattern. In our study [3], patients with low LDL-C and high non-HDL-C had higher levels of triglycerides than the other patients and high triglyceride levels have been related to discordance of LDL-C and non-HDL-C in previous studies [6, 7]. In the current study [1], there was no significant difference between patients with LDL-C levels within and above target regarding triglyceride levels.

Further studies should be designed to understand the demographic, clinical and laboratory characteristics of patients with discordant lipid parameters. Moreover, the prognosis of cardiovascular disease should be further investigated in follow-up studies in these patients. Despite the low levels of LDL-C, some patients still experience cardiovascular events and patients with low levels of LDL-C but with higher levels of other atherogenic lipid particles may need further evaluation, more aggressive management and close follow-up.

## Data Availability

Not applicable.

## Abbreviations

Apo B: Apolipoprotein B
LDL-C: low-density lipoprotein cholesterol
Non-HDL-C: non-high-density lipoprotein cholesterol
