# Correction to: Discordant serum lipid parameters

**DOI:** 10.1186/s12944-021-01469-x

**Published:** 2021-05-25

**Authors:** Ozge Kurmus, Turgay Aslan, Murat Eren, Kursat Akbuga

**Affiliations:** Department of Cardiology, Faculty of Medicine, Ufuk University, MevlanaBulvari (Konya yolu), No: 86-88, Balgat, Ankara, Turkey

**Correction to: Lipids Health Dis 20, 10 (2021)**

**https://doi.org/10.1186/s12944-021-01445-5**

Following publication of the original article [1], it was reported that the response by the authors of “Apolipoprotein B and non-high-density lipoprotein cholesterol reveal a high atherogenicity in individuals with type 2 diabetes and controlled low-density lipoprotein-cholesterol” [2] was missing in the main text. This response has been added and the original article has been corrected.
